# Sulfate assimilation regulates antioxidant defense response of the cyanobacterium *Synechococcus elongatus* PCC 7942 to high concentrations of carbon dioxide

**DOI:** 10.1128/aem.00115-25

**Published:** 2025-03-06

**Authors:** Yujie Mu, Huiting Chen, Jianwei Li, Pei Han, Zhen Yan

**Affiliations:** 1 Shandong Key Laboratory of Water Pollution Control and Resource Reuse, School of Environmental Science and Engineering, Shandong University 12589 , Qingdao, Shandong, China; 2 Key Laboratory of Space Utilization, Technology and Engineering Center for Space Utilization, Chinese Academy of Sciences 382688 , Beijing, China; 3 State Key Laboratory of Microbial Technology, Microbial Technology Institute, Shandong University State 214177 , Qingdao, Shandong, China; Georgia Institute of Technology, Atlanta, Georgia, USA

**Keywords:** cyanobacteria, carbon dioxide, oxidative stress, sulfate assimilation, metabolic regulation

## Abstract

The adaptive evolution of cyanobacteria over a prolonged period has allowed them to utilize carbon dioxide (CO_2_) at the low concentrations found in the atmosphere (0.04% CO_2_) for growth. However, whether the exposure of cyanobacteria to high concentrations of CO_2_ results in oxidative stress and the activation of antioxidant defense response remains unknown, albeit fluctuations in other culture conditions have been reported to exert these effects. The current study reveals the physiological regulation of the model cyanobacterium *Synechococcus elongatus* PCC 7942 upon exposure to 1% CO_2_ and the underlying mechanism. Exposure to 1% CO_2_ was demonstrated to induce oxidative stress and activate antioxidant defense responses in *S. elongatus*. Further analysis of variations in metabolism between *S. elongatus* cells grown at 0.04% CO_2_ and exposed to 1% CO_2_ revealed that sulfate assimilation was enhanced after the exposure to 1% CO_2_. A strain of *S. elongatus* lacking the gene *cysR*, encoding a global transcriptional regulator for genes involved in sulfate assimilation, was generated by deleting the gene from the genomic DNA. A comparative analysis of the wild-type and *cysR*-null strains indicated the regulation of the antioxidant response by sulfate assimilation. In addition, lines of evidence were presented that suggest a role of degradation of phycobilisome in the antioxidant response of *S. elongatus* under conditions of 1% CO_2_ and sulfate limitation. This study sheds light on the *in situ* effects of high CO_2_-induced stress on the ecophysiology of cyanobacteria upon exposure to diverse scenarios from a biotechnological and ecological perspective.

**IMPORTANCE:**

Cyanobacteria that grow autotrophically with CO_2_ as the sole carbon source can be subject to high-CO_2_ stress in a variety of biotechnological and ecological scenarios. However, physiological regulation of cyanobacteria in response to high-CO_2_ stress remains elusive. Here, we employed microbial physiological, biochemical, and genetic techniques to reveal the regulatory strategies of cyanobacteria in response to high-CO_2_ stress. This study, albeit physiological, provides a biotechnological enterprise for manipulating cyanobacteria as the chassis for CO_2_ conversion and sheds light on the *in situ* ecological effects of high CO_2_ on cyanobacteria.

## INTRODUCTION

Cyanobacteria originated on Earth approximately three billion years ago, leveraging light and carbon dioxide (CO_2_) as the energy and carbon sources, respectively, for oxygenic photosynthesis. Their origin represents a major milestone in the transition of the earth’s atmosphere from anaerobic to aerobic, often referred to as the Great Oxidation Event (1, 2). The gradual increase in the abundance of oxygen in the earth’s atmosphere accompanied the decrease in that of atmospheric CO_2_ from high (>1%) to low (~0.04%) levels (3, 4). This has resulted in a gradual and prolonged adaptive evolution of cyanobacteria to allow growth under conditions of low CO_2_ (5). For instance, the evolution of an effective strategy, known as the CO_2_-concentrating mechanism, allows for a marked improvement in the photoautotrophic performance of cyanobacteria under conditions of low CO_2_ (6, 7). Nevertheless, cyanobacteria can be subjected to high concentrations of CO_2_ in a variety of scenarios owing to the rapid increase in CO_2_ emissions as a result of anthropogenic and geological activities (8).

The rapid increase in the concentration of atmospheric CO_2_ has greatly contributed to global warming, and the large-scale emissions of CO_2_ are chiefly attributable to the massive use of fossil fuels (9). Various carbon capture, utilization, and storage technologies, as well as green and sustainable energy, have been developed to address this issue and reduce CO_2_ emissions. Given the relatively fast growth rate of cyanobacteria and their amenability to genetic manipulation, these microorganisms have been universally employed as an ideal chassis for the conversion of CO_2_ to high-value chemicals and sustainable biofuels; this process typically involves the culture of phototrophic cyanobacteria in conjunction with an elevated concentration of CO_2_ (10–13). On the other hand, cyanobacteria from natural habitats are inevitably exposed to high concentrations of CO_2_ under certain circumstances. For instance, the ecological impact of CO_2_ leakage from geological CO_2_ sequestration sites to aquatic and sedimentary environments has been widely acknowledged. Additionally, the impact of CO_2_ leakage on microbial activities has been demonstrated previously (14). Moreover, the ecological impact of CO_2_ emission from the shipping industry on aquatic (micro)organisms is becoming increasingly recognized (15). Therefore, acquiring an in-depth understanding of the physiological responses of cyanobacteria to high concentrations of CO_2_ is of great significance both from a biotechnological and an ecological perspective.

The effects of high levels of CO_2_ on the assimilation of nitrogen and phosphorus, as well as utilization of light by cyanobacteria, have been previously documented. In other words, the utilization of nitrogen, phosphorus, and light by cyanobacteria is enhanced under conditions of high CO_2_ (8). High levels of CO_2_ can lead to an imbalance in the metabolic pathways of cyanobacteria, thus stimulating the utilization of nitrogen and phosphorus for the synthesis of proteins, phospholipids, and adenosine triphosphate (ATP) (16). Limiting concentrations of nitrogen and phosphorus lead to the regulation of intracellular activities by the cyanobacteria and the degradation of additional cellular components, such as the phycobilisome, to compensate for the insufficiency of nitrogen and phosphorus (17). The utilization of light is also enhanced accordingly, thereby promoting photophosphorylation and generating more ATP and redox equivalents for promoting the metabolism of nitrogen and phosphorus. In addition, certain distinct functions of cyanobacteria, such as nitrogen fixation and toxin production, have also been reported to be enhanced with augmented energy metabolism under high concentrations of CO_2_ (8).

In general, external stress conditions, such as high salinity, light fluctuations, or nutrient deficiencies, inevitably lead to the generation of intracellular reactive oxygen species (ROS) in cyanobacteria due to the stress-induced imbalance between light-driven electron transport and the consumption of electrons for CO_2_ fixation (18, 19). This resulting ROS is sensed by the cyanobacteria, leading to the rapid initiation of antioxidant responses, including the synthesis of the antioxidant enzymes superoxide dismutase, catalases, and peroxidases, to scavenge the ROS (20–22). Thus, the antioxidant responses are crucial for cyanobacterial survival and growth when subjected to external stress conditions. However, whether high levels of CO_2_ can be regarded as an oxidative stress for cyanobacteria, which activates antioxidant responses, remains unclear. To address this enigmatic question, a model cyanobacterial strain, *Synechococcus elongatus* PCC 7942 (hereafter referred to as *S. elongatus* PCC 7942), was cultured initially under conditions of atmospheric CO_2_ and then transferred to an environment with high CO_2_ concentrations (1% CO_2_). The physiological characteristics of cyanobacteria upon exposure to 1% CO_2_ were characterized by various physiological, biochemical, and genetic methods. This approach ultimately revealed the physiological regulation of *S. elongatus* PCC 7942 in response to high levels of CO_2_ and the underlying strategies.

## MATERIALS AND METHODS

### Cyanobacterial strains and growth conditions

*S. elongatus* PCC 7942 (wild type [WT]) and its mutants were cultured in BG-11 medium (100 mL volume) and incubated under rotary conditions (120 rpm) at 30°C under constant illumination with light of 50 µmol photon/m^2^/s. To investigate the physiological responses of *S. elongatus* PCC 7942 upon exposure to 1% CO_2_, the strain was initially cultured under conditions of atmospheric (0.04%) CO_2_ until an optical density at 730 nm (OD_730_) of 0.8 was attained; the cells were subsequently transferred to an atmosphere containing 1% (vol/vol) CO_2_. BG-11 medium lacking sulfate was obtained by replacing magnesium sulfate (MgSO_4_) with an equivalent amount of magnesium chloride (MgCl_2_) (23). Sulfate-replete BG-11 medium containing 1, 20, 40, 100, 100, 200, or 300 µM of sulfate was obtained by adding the corresponding concentrations of MgSO_4_. The cultures (2 mL) were extracted from screw-capped flasks every 2 days, and cell growth was monitored via a spectrophotometer (METASH UV-6100, China) by measuring OD_730_.

### Construction of mutant strains of *S. elongatus* PCC 7942

The *ΔgpxA* and *ΔcysR* strains of *S. elongatus* PCC 7942, which contain null mutations in the genes *gpxA* and *cysR*, were generated via plasmid construction and natural transformation (24). A gene knockout vector containing 1,000 bp DNA sequences flanking the target genes *gpxA* and *cysR* and a gene encoding spectinomycin resistance was assembled using plasmid pUC19 as the skeleton. The resultant recombinant plasmids were amplified and isolated from *Escherichia coli* DH5α. Recombinant plasmids (100 ng) were added to *S. elongatus* PCC7942 and cultured for 17 h at 30°C away from light. Following transformation, the strains were cultured on BG-11 plates supplemented with 2 µg/mL each of spectinomycin and streptomycin. The BG-11 plates employed for solid plate culture additionally contained 1% (wt/vol) agarose (Biosharp, China), 20 mM sodium bicarbonate (NaHCO_3_), and 1.6 mM sodium thiosulfate (Na_2_S_2_O_3_); these were incubated at 30°C in the presence of light of 50 µmol photon/m^2^/s. Deletion of the *gpxA* and *cysR* genes was confirmed via colony polymerase chain reaction (PCR) and sequencing. The PCR reaction conditions included initial denaturation at 95°C for 5 min, followed by 35 cycles of denaturation at 94°C for 25 s, annealing at 55℃ for 40 s, and extension at 72°C for 45 s, and finally, heat preservation at 72°C for 10 min. The sequence of primers is shown in Table S5.

### Real-time quantitative PCR analysis

Real-time quantitative PCR (RT-qPCR) analysis was employed for quantifying the abundance of key differentially expressed genes (DEGs) for validating the results obtained from transcriptomic analysis. The cells from the WT strain of *S. elongatus* PCC7942 and its mutants, Δ*gpxA* and Δ*cysR*, were sampled. Total RNA was isolated from log-phase cultures of cyanobacteria using the M5 EASYspin Plus Bacterial RNA Rapid Extraction Kit (Mei5bio, Beijing, China). After quantification, the extracted RNA was reverse-transcribed into cDNA using the M5 Super plus qPCR RT kit with gDNA remover (Mei5bio, Beijing, China), and stored at −20°C. Following reverse transcription, a 20 µL PCR reaction system consisting of the cDNA template, primer solutions, and SYBR Premix EsTaq (with Tli RNaseH) (Mei5bio, Beijing, China) was prepared with 2.0, 0.8, and 17.2 µL, respectively, of the components. All procedures were performed under ice-cold and dark conditions. 16S rDNA was employed as the internal control, and each sample was evaluated in triplicate for consistency. The samples were analyzed using a 7900HT Fast Real-Time PCR System (Thermo Fisher Scientific, Waltham, USA). The sequence of primers is shown in Table S6. The fluorescence quantitative PCR conditions for RT-qPCR included initial denaturation at 95°C for 30 s, followed by 40 cycles of denaturation at 94°C for 15 s, annealing at 55°C for 30 s, and extension at 72°C for 60 s. The target genes were quantified by the 2^−ΔΔCt^ method. The formula was as follows:


$$-\Delta \Delta Ct=[({Ct}_{\text{ target gene }}-{Ct}_{\text{ internal control}}{)}_{\text{ control }}-({Ct}_{\text{ target gene }}-{Ct}_{\text{ internal control}}{)}_{\text{ sample}}],$$

where Ct is the cycle threshold.

### Analysis of oxidative stress and antioxidant responses of *S. elongatus* PCC 7942

The concentrations of ROS and total reduced thiol pool levels were measured using respective assay kits (Nanjing Jiancheng Bioengineering Institute, Nanjing, China) according to the manufacturer’s guidelines (18). Cyanobacterial culture (2 mL) was centrifuged at 8,000 × *g* for 5 min. The supernatant was discarded, and the cells were resuspended in 10 mM phosphate-buffered saline (PBS, pH 7.2 and 7.4) to a concentration of 1 × 10^8^ cells/ml. Intracellular ROS was detected by the DCFH-DA fluorescent probe, and fluorescence detection was carried out by a fluorescence microplate reader (Varioskan Lux; Thermo Fisher Scientific). For the total reduced thiol pools assay, a 5 mL cell suspension was prepared for ultrasonication (ultrasonic power 100 W, working for 5 s, interval of 5 s, and total crushing for 10 min). The reaction of reduced thiol pools with 5,5′-dithiobis-2-nitrobenoic acid yields a yellow compound, which was subsequently quantified by colorimetry at 405 nm.

### Measurement of the contents of phycocyanin and allophycocyanin

Cyanobacterial culture (2 mL) was withdrawn at the stipulated intervals (2 days after the transfer to the atmosphere with 1% CO_2_) and centrifuged at 8,000 × *g* for 5 min. The supernatant was discarded, and the pellet was resuspended in 10 mL PBS (pH 7.2 and 7.4). The cell suspension was subjected to ultrasonication as detailed above, followed by centrifugation at 10,000 × *g* for 5 min at 4°C. The content of phycocyanin (PC) and allophycocyanin (APC) in the supernatant was quantified by determining absorbance at 620 and 650 nm, respectively, using a UV-vis spectrophotometer at 620 and 650 nm for PC and APC, respectively (25).

### Transcriptome sequencing and bioinformatics analyses

To investigate the responses of *S. elongatus* PCC 7942 upon exposure to 1% CO_2_, a culture of the strain grown to an OD_730_ of 0.8 was transferred to an atmosphere containing 1% CO_2_ for durations of 40 min or 4 h. The cells were then centrifuged at 10,000 × *g* for 10 min. A similar procedure was followed for investigating the effects of sulfate limitation on WT and Δ*cysR* strains, wherein the cultures were grown to an OD_730_ of 0.8, transferred to an atmosphere containing 1% CO_2_ for 4 h, and then centrifuged. The experiment was conducted using biological duplicate samples. Total RNA was extracted from *S. elongatus* using TRIzol Reagent, and genomic DNA was eliminated using DNase I (Takara, Japan). Total RNA (5 µg) from each sample was utilized as a starting material for generating sequencing libraries using the TruSeq RNA sample preparation kit (Illumina, San Diego, CA, USA). The paired-end libraries so obtained were sequenced using an Illumina NovaSeq 6000 sequencing instrument (BIOZERON Co., Ltd., Shanghai, China).

Gene reads were sourced from Rockhopper (https://cs.wellesley.edu/~btjaden/Rockhopper/). Gene expression data were derived via local regression analysis, followed by a null hypothesis test using the negative binomial model for each gene comparison, yielding the corresponding *P* values. The Benjamini-Hochberg (BH, a method for controlling false discovery rate [FDR]) multiple modified *P* values were employed for calculating the FDR for significant gene comparisons. The selection criteria for DEGs were based on log_2_(fold change), FDR, and additional parameters. Log_2_(fold change) values of ≥1 and FDR of ≤0.05 served as the screening thresholds for characterizing DEGs. The functions of DEGs were elucidated by Kyoto Encyclopedia of Genes and Genomes (KEGG) pathway analysis using KOBAS (http://bioinfo.org/kobas/) and Fisher’s exact test for statistical calculation. The BH (FDR) method was employed for performing multiple tests to control the identification of false positives. The calculation formula was the same as in the previous section, and the *P* value of 0.05 was set as the threshold. The KEGG pathways that met this condition were defined as the pathways that were significantly enriched in DEGs. The genome annotation used for all analyses and primer design was from KEGG and is available at https://www.genome.jp/kegg-bin/show_organism?org=syf.

### Statistical analyses

Statistical analyses were carried out using Origin. Statistical significance of comparison among groups was determined using Student’s *t*-test. The differences were considered statistically significant at *P* < 0.05, indicated by *, **, and *** for *P* < 0.05, *P* < 0.01, and *P* < 0.001, respectively.

## RESULTS

### Exposure to 1% CO_2_ activated antioxidant defense responses in *S. elongatus* PCC 7942

**Fig 1:**
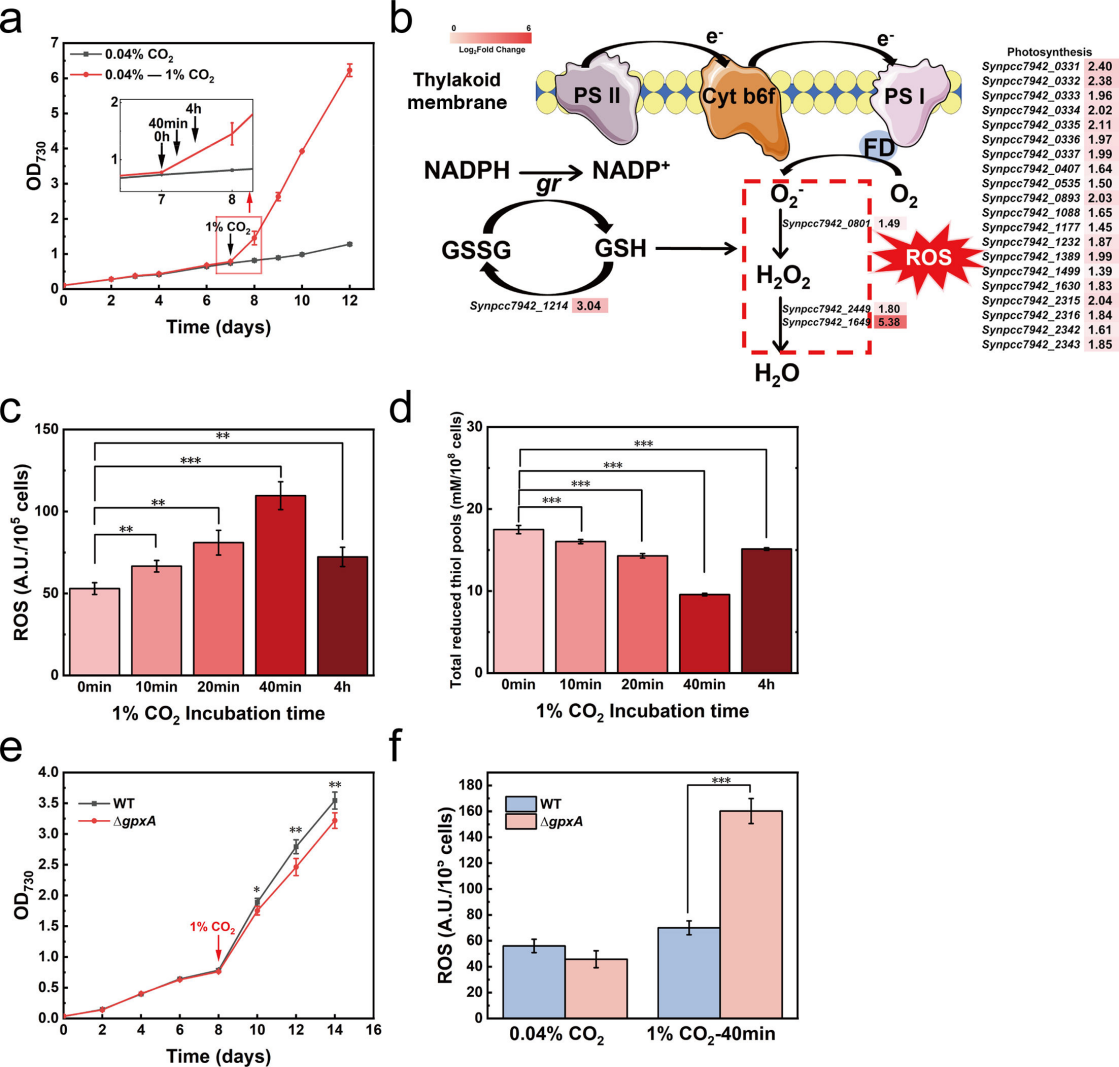
Exposure to 1% CO_2_ activated antioxidant defense responses in *S. elongatus* PCC 7942. (a) Growth curve of *S. elongatus* PCC 7942 upon exposure to 0.04% and 1.0% CO_2_. The arrow indicates the time points of culture under conditions of 0.04% CO_2_ and then transferred to 1% CO_2_. (b) Metabolic pathway for the generation of oxidative stress and antioxidant defense responses in *S. elongatus* PCC 7942. The significant differentially expressed genes are represented in the heat map. (c) Intracellular levels of ROS exposed to 1% CO_2_. (d) Intracellular levels of total reduced thiol pools exposed to 1% CO_2_. (e) Growth curve of WT strain and a *gpxA*-null mutant (∆*gpxA*) exposed to 0.04% and 1% CO_2_. The arrow indicates the time point transferred to 1% CO_2_. (f) Intracellular levels of ROS of WT and ∆*gpxA* exposed to 0.04% CO_2_ and exposed to 1% CO_2_ for 40 min. The differences were considered significant at *P* < 0.05 and were referred to as *P < 0.05, **P < 0.01, and ***P < 0.001. Abbreviations: FD, ferredoxin; *gr*, glutathione reductase; GSH, glutathione; GSSG, glutathione disulfide; ROS, reactive oxygen species; *Synpcc7942_1214*, glutathione peroxidase; *Synpcc7942_0801*, superoxide dismutase; *Synpcc7942_2449*, peroxiredoxins; *Synpcc7942_1649*, rubrerythrin; WT, wild type.

*S. elongatus* PCC 7942 was initially cultured under conditions of atmospheric CO_2_ (~0.04% CO_2_) until an OD_730_ of 0.8 was attained. The culture was subsequently transferred to an atmosphere containing 1% CO_2_ without changing other culture conditions. Note that exposure to 1% CO_2_ lowered the pH of the culture from ~11 to ~7 within 4 h (Fig. S1). The photoautotrophic growth of *S. elongatus* PCC 7942 was significantly augmented after the transfer to 1% CO_2_ (Fig. 1a). The cells of *S. elongatus* PCC 7942 were sampled during the initial growth under 0.04% CO_2_ and 40 min and 4 h after the transfer to 1% CO_2_ and were analyzed by transcriptome sequencing. A total of 477 and 654 genes were significantly upregulated in the cells exposed to 1% CO_2_ for 40 min and 4 h, respectively, compared to those in the cells grown under conditions of 0.04% CO_2_ (Fig. S2). KEGG analysis indicated that 30% and 57% of the upregulated genes in cells exposed to 1% CO_2_ for 40 min and 4 h, respectively, were enriched in energy and biosynthetic metabolisms, including photophosphorylation, Calvin cycle, and macromolecular biosynthesis, a result consistent with the observed augmentation in growth after transfer to 1% CO_2_ (Fig. S3; Table S2 and S3).

Notably, the expression of genes encoding enzymes involved in antioxidant defense responses, such as *Synpcc7942_0801* encoding superoxide dismutase and *Synpcc7942_1214*, *Synpcc7942_2309* and *Synpcc7942_2449* encoding peroxidases, was significantly upregulated upon exposure of *S. elongatus* PCC 7942 to 1% CO_2_ for 40 min compared to the cells grown at 0.04% CO_2_. This observation indicates that short-term exposure of *S. elongatus* PCC 7942 to 1% CO_2_ may have induced oxidative stress, thereby activating the antioxidant defense responses (Fig. 1b; Table S1). Considering the rapid decrease in pH of the culture due to exposure to 1% CO_2_, in order to determine whether the oxidative stress was induced by 1% CO_2_ or pH decrease, we measured ROS abundances upon exposure of the culture to 1% CO_2_ and ROS abundances upon pH adjustment of the culture with HCl, which was comparable to the pH decrease caused upon exposure to 1% CO_2_. No significant change in ROS abundance was observed upon pH adjustment of the culture with HCl (Fig. S4), while a significant increase in ROS abundance was observed upon exposure of the culture to 1% CO_2_ (Fig. 1c), suggesting that the oxidative stress was induced by 1% CO_2_ independent of a change in pH. Moreover, a gradual increase in the concentration of ROS concomitantly with a decrease in the levels of total reduced thiol pools was observed upon exposure of the cells to 1% CO_2_ for 40 min (Fig. 1c and d). The decrease in the levels of total reduced thiol pools may be attributed to the increased oxidation of GSH to glutathione disulfide (GSSG) accompanying ROS scavenging; this reaction is catalyzed by glutathione peroxidase encoded by the gene *Synpcc7942_1214* (*gpxA*), whose expression in the cells exposed to 1% CO_2_ for 40 min was upregulated nearly threefold compared to that in the cells sampled at 0.04% CO_2_ (Fig. 1b). Therefore, the gene *gpxA* was deleted in *S. elongatus* PCC7942 to generate the ∆*gpxA* mutant strain. A slower growth rate and increased levels of ROS were observed upon exposure of the mutant strain to 1% CO_2_ compared with the corresponding values for the WT strain (Fig. 1e and f); this is attributable to the inhibition of GSH oxidation to GSSG in the ∆*gpxA* strain (26), thereby aggravating the oxidative stress under conditions of 1% CO_2_. Taken together, these results demonstrate that exposure to 1% CO_2_ induces oxidative stress and activates antioxidant defense responses in *S. elongatus* PCC 7942.

### Antioxidant defense responses in *S. elongatus* PCC 7942 exposed to 1% CO_2_ are regulated by sulfate assimilation

**Fig 2:**
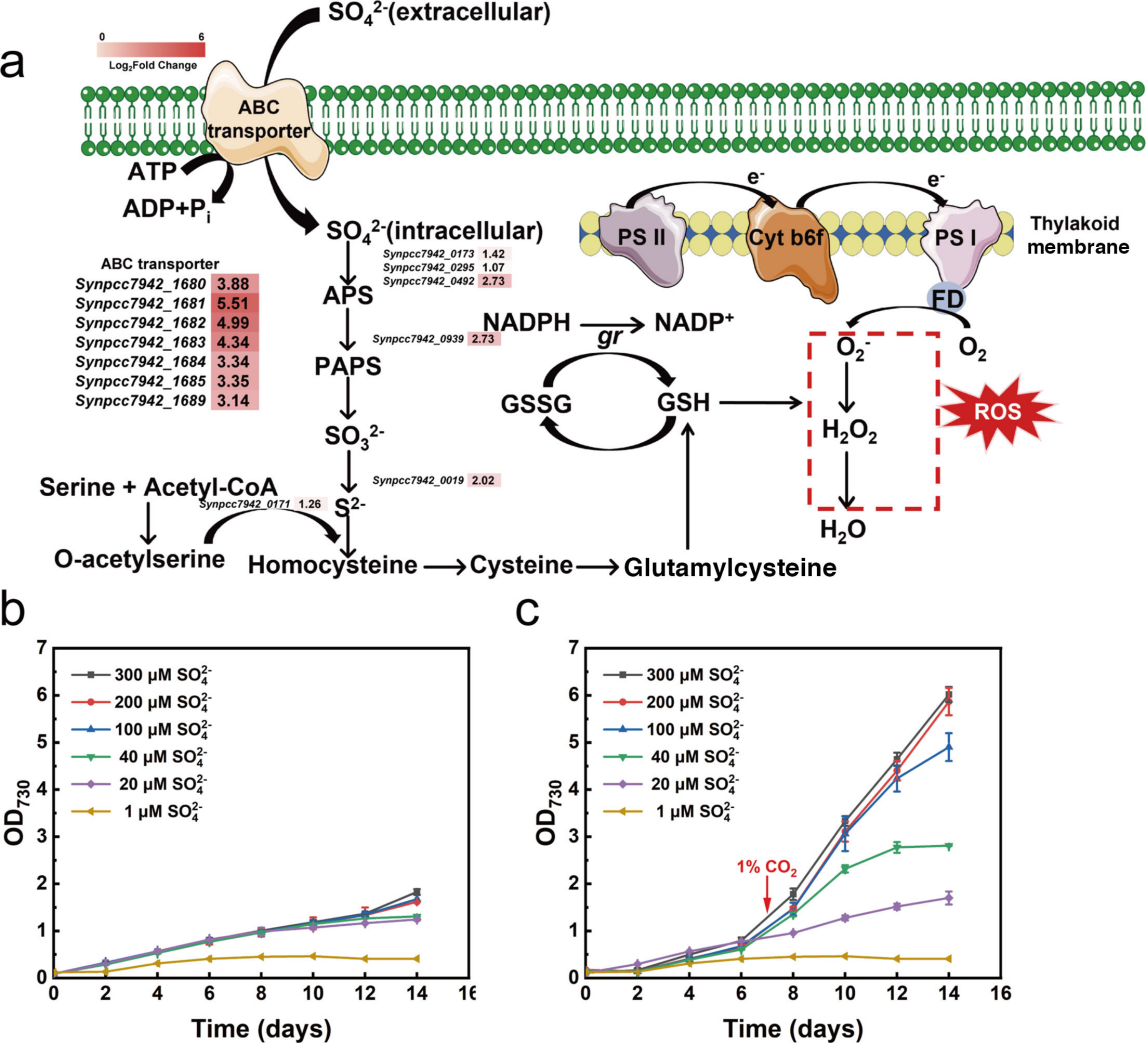
Sulfate assimilation was enhanced in *S. elongatus* PCC 7942 culture exposed to 1% CO_2_. (a) Cyanobacterial metabolic pathway of sulfate assimilation. The significant differentially expressed genes are represented in the heat map. (b) Growth curve of *S. elongatus* PCC 7942 cultured with different concentrations of sulfate exposed to 0.04% CO_2_. (c) Growth curve of *S. elongatus* PCC 7942 cultured with different concentrations of sulfate under conditions of 0.04% CO_2_ and then transferred to 1% CO_2_. The arrow indicates the time point of culture being transferred to 1% CO_2_. Abbreviations: *Synpcc7942_0173*, 3′-phosphoadenosine 5'-phosphosulfate synthase; *Synpcc7942_0295*, sulfate adenylyltransferase; *Synpcc_0492*, bifunctional enzyme; *Synpcc7942_0939*, APS kinase, *Synpcc_0019,* sulfite reductase (ferredoxin), *Synpcc_*0171, cysteine synthase.

The comparative analysis of the transcriptome of *S. elongatus* PCC 7942 upon exposure to 1% CO_2_ for 40 min unexpectedly identified several DEGs that are involved in sulfate assimilation; for instance, genes encoding proteins that play a role in sulfate transport (*Synpcc7942_1680* encoding *cysA*, *Synpcc7942_1687* encoding *cysT*, *Synpcc7942_1684* encoding *cysR*, etc.) and pathways of sulfate reduction (*Synpcc7942_0295* encoding *sat*, *Synpcc7942_0939* encoding *cysC*, *Synpcc7942_0492* encoding bifunctional enzyme, etc.) were significantly upregulated in the cells exposed to 1% CO_2_ for 40 min compared to those in the cells sampled under 0.04% CO_2_ (Fig. 2a; Table S1). RT-qPCR analysis further confirmed that the significant upregulation of these genes involved in sulfate assimilation was due to exposure of the cells to 1% CO_2_ but not due to pH decrease (Fig. S5). To evaluate the role of sulfate assimilation in cells exposed to 1% CO_2_, *S. elongatus* PCC 7942 was cultured in media containing different concentrations of sulfate and incubated under conditions of 0.04% CO_2_; the cells were subsequently either transferred to the atmosphere with 1% CO_2_ or maintained at 0.04% CO_2_. Figure 2b and c show that the presence of 20–300 µM of sulfate did not impact the growth of *S. elongatus* PCC 7942 under conditions of 0.04% CO_2_; however, a dose-dependent stimulatory effect on the growth of *S. elongatus* PCC 7942 was observed with the same concentrations of sulfate upon transfer to 1% CO_2_. This observation indicates that sulfate is essential for maintaining the growth of *S. elongatus* PCC 7942 under conditions of 1% CO_2_. Given that cysteine, the product of sulfate assimilation, is a key component of GSH that plays an important role in scavenging ROS, we speculated that the activation of the antioxidant defense responses may be regulated by sulfate assimilation under conditions of 1% CO_2_ (27).

**Fig 3:**
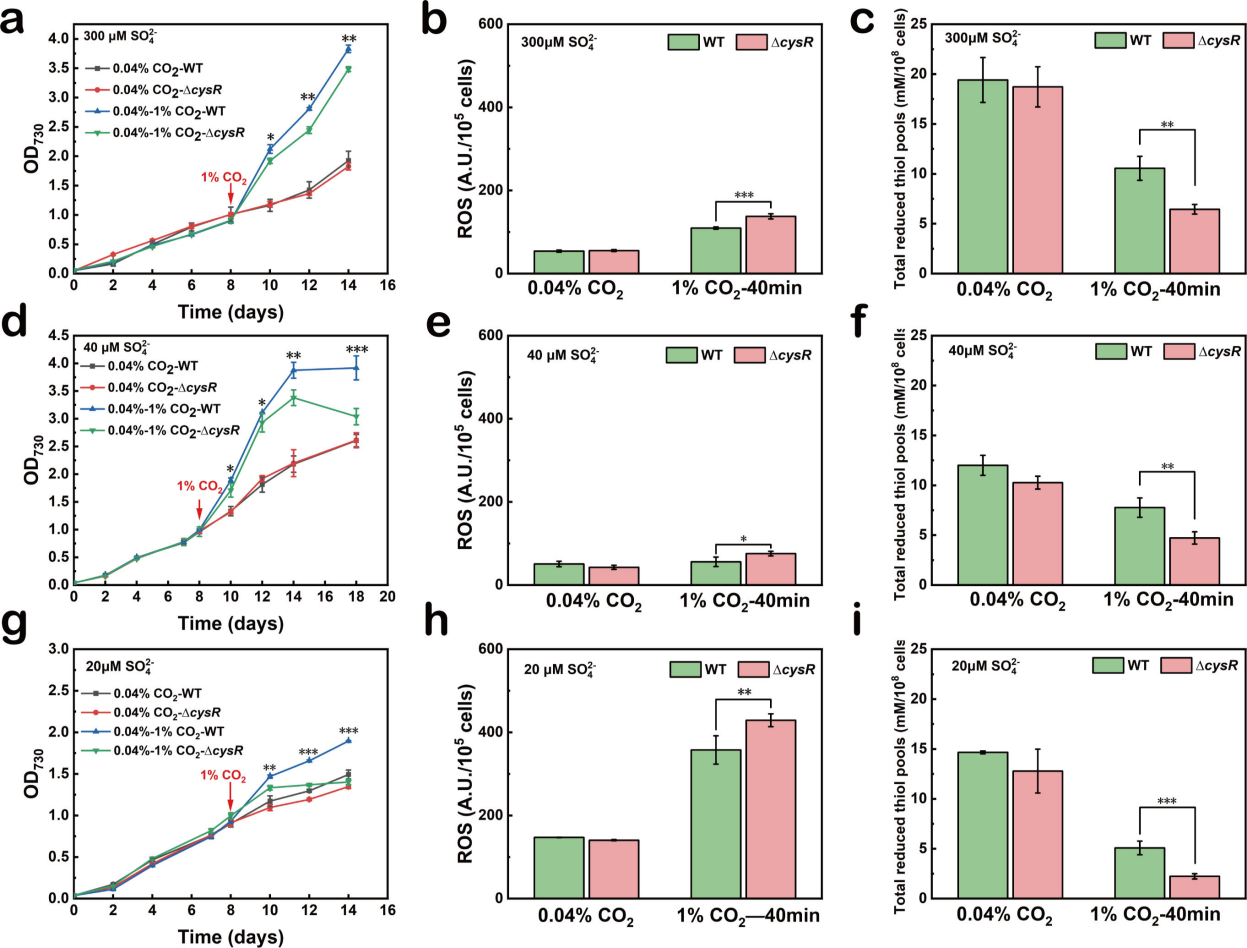
Antioxidative defense responses of *S. elongatus* PCC 7942 exposed to 1% CO_2_ are regulated by sulfate assimilation. Growth curves of wild-type (WT) and a cysR-null mutant (∆*cysR*) under different CO_2_ levels and the sulfate concentrations at 300 µM (a), 40 µM (d), and 20 µM (g). The arrows indicate the time point of culture being transferred to 1% CO_2_. Intracellular levels of reactive oxygen species (ROS) of WT and ∆*cysR* exposed to 0.04% and 1% CO_2_ with the sulfate concentrations at 300 µM (b), 40 µM (e), and 20 µM (h). Intracellular levels of total reduced thiol pools of WT and ∆*cysR* exposed to 0.04% and 1.0% CO_2_ with the sulfate concentrations at 300 µM (c), 40 µM (f), and 20 µM (i). The differences were considered significant at *P* < 0.05 and were referred to as *P < 0.05, **P < 0.01, and ***P < 0.001.

The cyanobacterial sulfate assimilatory pathway involves the uptake of sulfate and its intracellular reduction (28, 29). The uptake of sulfate by cyanobacteria is known to be mediated by an ABC-type transporter complex that is encoded by the *cys* operon. The gene *Synpcc7942_1684* encoding *cysR* of the *cys* operon was previously identified to encode a transcription factor that regulates the expression of genes encoding sulfate transporters (29). To further dissect the regulatory role of sulfate assimilation under conditions of 1% CO_2_, a *cysR*-null strain (∆*cysR*) of *S. elongatus* PCC 7942 was constructed by knocking out *cysR* from the genomic DNA. The WT and ∆*cysR* strains of *S. elongatus* PCC 7942 were cultured with different concentrations of sulfate under conditions of 0.04% CO_2_ and then transferred to 1% CO_2_. Figure 3a, d, and g reveal the absence of differences in growth between the WT and ∆*cysR* strains under conditions of 0.04% CO_2_, while the growth of the WT strain was significantly better than that of the ∆*cysR* strain following the transfer to 1% CO_2_ under sulfate-sufficient (300 µM) as well as sulfate-limiting (40 or 20 µM) conditions. The differences in the growth of the WT and ∆*cysR* strains under conditions of 1% CO_2_ are attributable to variations in the concentrations of intracellular ROS and total reduced thiol pools. Figure 3b through i reveal the absence of significant variation in the concentrations of ROS and total reduced thiol pools under conditions of 0.04% CO_2_, while exposure of the cells to 1% CO_2_ for 40 min resulted in lower levels of ROS and greater concentration of total reduced thiol pools in the WT strain compared to those in the ∆*cysR* strain. This observation is reflective of a higher degree of antioxidant activities in the WT strain compared to those in the ∆*cysR* strain under conditions of 1% CO_2_. Given that *cysR* is a global regulator of sulfate assimilation, these results suggest that antioxidant defense responses in *S. elongatus* exposed to 1% CO_2_ are regulated by sulfate assimilation.

### Phycobilisomes were observed to be degraded in *S. elongatus* PCC 7942 under conditions of 1% CO_2_ and sulfate limitation

**Fig 4:**
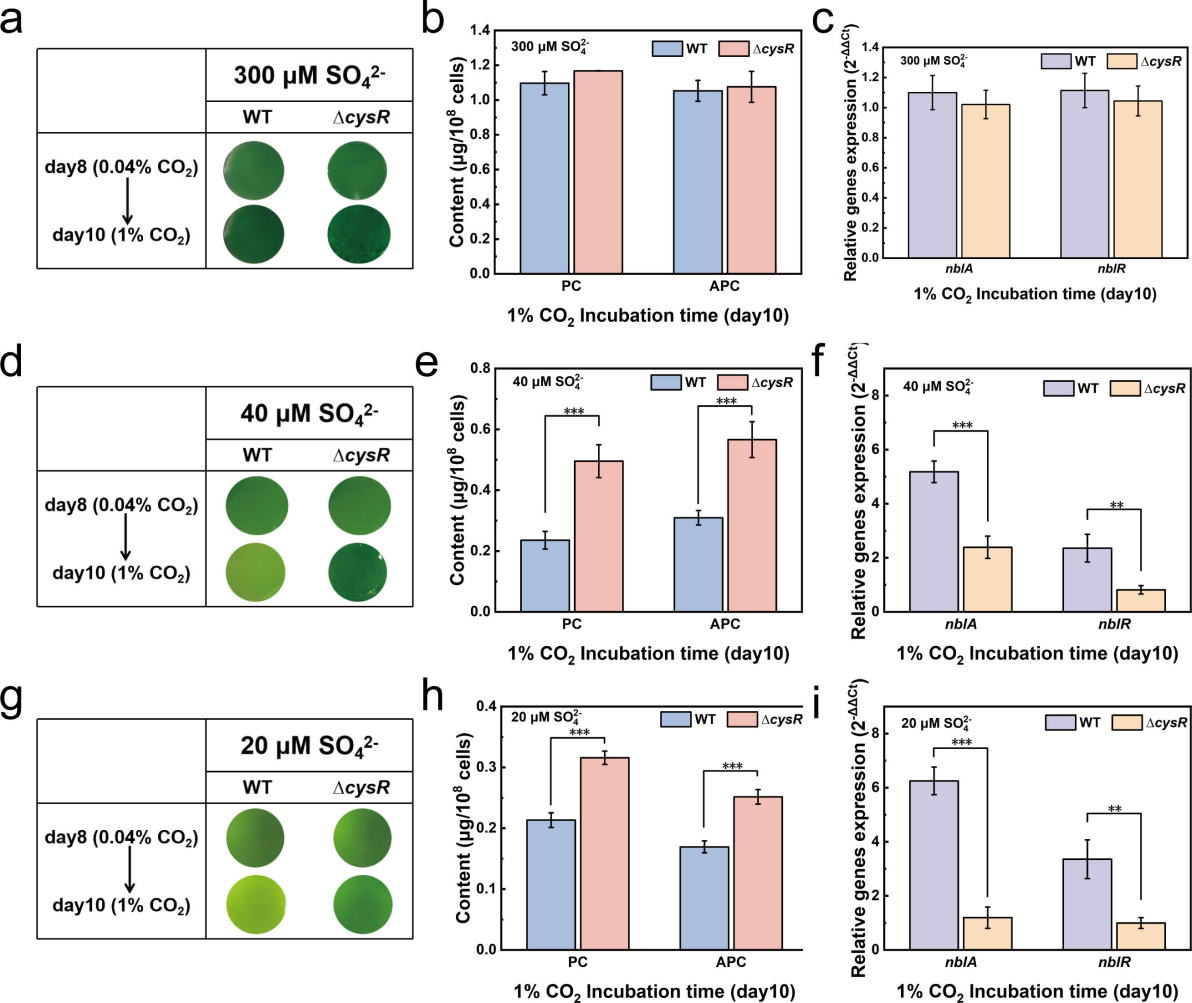
Degradation of phycobilisome assists the growth and antioxidant defense responses of *S. elongatus* PCC 7942 under conditions of 1% CO_2_ and sulfate limitation. The cultures of WT and Δ*cysR* were cultured under conditions of 0.04% CO_2_ and then transferred to 1% CO_2_ on day 8. Representative appearance under conditions of 0.04% CO_2_ on day 8 and then transferred to 1% CO_2_ on day 10 at the sulfate concentrations of 300 µM (a), 40 µM (d), and 20 µM (g). Phycobilin (PC) and allophycocyanin (APC) on day 10 at the sulfate concentrations of 300 µM (b), 40 µM (e), and 20 µM (h). Relative gene expression transferred to 1% CO_2_ on day 10 compared to the condition of 0.04% CO_2_ on day 8 at the sulfate concentrations of 300 µM (c), 40 µM (f), and 20 µM (i). The differences were considered significant at *P* < 0.05 and were referred to as**P < 0.01 and ***P < 0.001.

Interestingly, a phenotypic change was observed in cells of the WT strain grown under sulfate-limiting conditions (20 or 40 µM) 2 days after their transfer from 0.04% to 1% CO_2_. The color of cells of the WT strain changed from blue-green to yellow-green, a phenomenon termed bleaching, while a similar phenotypic change was not observed for the ∆*cysR* strain under identical conditions (Fig. 4a, d, and g). The degradation of phycobilisomes under nutrient-limiting conditions has been previously reported to result in the bleaching phenotypes (30, 31). The two known phycobilisomes in *S. elongatus* PCC 7942, PC and APC, were therefore quantified. Figure 4b, e, and h show that the abundance of PC and APC in the WT strain was significantly lower compared to those in the ∆*cysR* strain under conditions of 1% CO_2_ and sulfate limitation; however, significant differences between the WT and ∆*cysR* strains were not observed under conditions of 1% CO_2_ and sulfate sufficiency.

To gain better insights into the physiological differences between the WT and ∆*cysR* strains under conditions of 1% CO_2_ and sulfate limitation, the differences in gene expression profile between the two strains were analyzed via RT-qPCR and transcriptome sequencing (Fig. S6 and S7). Figure 4c, f, and i show that the expression of two genes *nblA* encoded by the gene *Synpcc7942_2127* and *nblR* encoded by the gene *Synpcc7942_2305*, which play a role in regulating the degradation of phycobilisomes (32, 33), was significantly upregulated in the WT strain compared to that in ∆*cysR*, which is consistent with the observed degradation of phycobilisomes in the WT strain under conditions of 1% CO_2_ and sulfate limitation. In addition, the expression of certain genes involved in photosynthesis and anabolism was significantly upregulated in the WT strain compared to that in the ∆*cysR* strain (Table S4), which was consistent with the better growth phenotype of the WT strain compared to that of the ∆*cysR* strain under the aforementioned conditions (Fig. 3a, d, and g). Additionally, the expression of key genes involved in antioxidant defense responses, including *gpxA* encoded by the gene *Synpcc7942_1214* and *prx* encoded by the gene *Synpcc7942_2449*, was significantly upregulated in the WT strain compared to that in the ∆*cysR* strain (Table S4), which was consistent with the measured differences in the concentrations of ROS and total reduced thiol pools between the WT and ∆*cysR* strains under the conditions of 1% CO_2_ and sulfate limitation (Fig. 3b, e, and h). Taken together, these results suggest that the degradation of phycobilisomes plays a role in the growth and antioxidant defense response of *S. elongatus* PCC 7942 under the aforementioned conditions of 1% CO_2_ and sulfate limitation.

## DISCUSSION

Given the current lack of clear standards as to what constitutes a high concentration of CO_2_, the present work utilized 1% CO_2_ to represent conditions of high CO_2_; this concentration is approximately 25-fold higher than that of atmospheric CO_2_. Moreover, previous studies have considered 1% or higher concentrations of CO_2_ to represent high levels. For instance, several reports on the utilization of cyanobacteria as cell factories employed 1%–5% CO_2_ as the carbon source and feedstock for enhancing cyanobacterial growth and the yield of high-value chemicals (34–39). In addition, 1% CO_2_ was employed in a study on the ecological impact of leakage of CO_2_ from geological CO_2_ storage sites on groundwater and soil (14). However, investigations of the ecological impact of emissions from industrial or shipping sources often employ 10% or higher concentrations of CO_2_ to represent high CO_2_ levels (15). Overall, the 1% CO_2_ concentration employed in the current study seeks to balance considerations and impacts from the perspective of both biotechnology and ecology.

ROS are inevitably generated in photoautotrophs such as cyanobacteria as the rate of electron generation via the photosynthetic electron transport chain exceeds the rate of electron consumption for CO_2_ fixation (20, 40). The imbalance between the production and consumption of electrons is mainly due to fluctuations in growth conditions. For example, fluctuations in the intensity of light, which is an essential energy source for the growth of cyanobacteria, were first identified as a causative factor for oxidative stress (20). A light intensity that exceeds the capacity of the photosynthetic electron transport chain leads to the passive utilization of molecular oxygen as the final electron acceptor in the electron transport chain, resulting in the generation of superoxide anions and hydrogen peroxide (41, 42). Subsequently, fluctuations in salinity, temperature, or nitrogen source were also identified as causative factors of oxidative stress, albeit the pathways that perceive fluctuations in these conditions appear to vary (18, 19, 43). The current work is the first to report that fluctuations in the concentration of CO_2_, the sole carbon source for the growth of the cyanobacterium *S. elongatus* PCC 7942, caused oxidative stress (Fig. 1). Transcriptome analysis indicated that exposure to 1% CO_2_ greatly altered the metabolic profiles of *S. elongatus* PCC 7942. As expected, a significant upregulation of the genes involved in photophosphorylation that is responsible for electron generation was observed (Fig. 1; Table S2 and S3). Moreover, the expression of genes involved in the biosynthetic metabolism of macromolecules such as amino acids, fatty acids, or pigments was also significantly upregulated (Fig. S3), which are responsible for electron consumption. As a result, the key requirements for the generation of ROS, i.e., imbalance between electron generation and consumption, are met. Therefore, the exposure of *S. elongatus* PCC 7942 to high concentrations of CO_2_ resulted in oxidative stress.

To cope with the oxidative stress induced by high CO_2_, the antioxidant defense response was expectedly activated in *S. elongatus* PCC 7942. Deletion of the gene *gpxA* encoding glutathione peroxidase, which catalyzes the oxidation of GSH for scavenging ROS, significantly aggravated oxidative stress and negatively affected the growth of *S. elongatus* PCC 7942 under conditions of 1% CO_2_ (Fig. 1e and f). In addition to the antioxidant defense pathways, sulfate assimilation was unexpectedly enhanced in *S. elongatus* PCC 7942 subjected to conditions of 1% CO_2_. Further, the growth of *S. elongatus* PCC 7942 under conditions of 1% CO_2_ was dependent on sulfate concentration over the range 20–300 µM (Fig. 2). Given that the product of sulfate assimilation, cysteine, is one of the components of the antioxidant GSH, we speculated that the enhanced sulfate assimilation was likely to be closely related to the antioxidant response under high CO_2_ level. This speculation was supported by the fact that there were less ROS abundances and more reduced thiol pools in the sulfate-rich condition (300 µM) compared to the sulfate-limited condition (20 µM) (Fig. 3b and h). Further deletion of the gene *cysR* encoded by the gene *Synpcc7942_1684* that regulates sulfate assimilation indicates that the oxidative stress was significantly aggravated and the antioxidative response was weakened compared to the WT strain under the high-CO_2_ and sulfated-limited conditions (Fig. 3). Therefore, a regulatory mechanism of sulfate assimilation on the antioxidative response under high-CO_2_ stress is for the first time established in cyanobacteria.

In addition to sulfate assimilation, degradation of phycobilisomes was unexpectedly observed in the WT strain but not in the ∆*cysR* strain under the aforementioned conditions of high CO_2_ and sulfate limitation, suggesting a role of CysR in regulating the degradation of phycobilisome (Fig. 4). The regulatory mechanism of phycobilisome degradation under nitrogen-limiting conditions has been extensively studied (30, 32, 33, 44, 45). The transcription factor NblR (encoded by the gene *Synpcc7942_2305*), which is activated under conditions of nutrient starvation, activates the transcription of *nblA* (encoded by the gene *Synpcc7942_2127*) that encodes a phycobilisome-degrading enzyme, thereby initiating the degradation of phycobilisome (33). It has also been reported that the transcription of *nblA* (encoded by the gene *Synpcc7942_2127*) is also regulated by NtcA (encoded by the gene *Synpcc7942_0127*) that is a global regulator under nitrogen-starved stress (46, 47). Additionally, the transcription of *nblA* (encoded by the gene *Synpcc7942_2127*) is reportedly regulated by NtcA (encoded by the gene *Synpcc7942_0127*), a global regulator under conditions of nitrogen starvation–induced stress (47). Constitutively expressed NblR (encoded by the gene *Synpcc7942_2305*) has been demonstrated to function in conjunction with NtcA (encoded by the gene *Synpcc7942_0127*) and activate the expression of *nblA* (encoded by the gene *Synpcc7942_2127*) by binding its promoter region under conditions of nitrogen starvation. This process was hitherto believed to be specific to nitrogen starvation-induced stress, and its occurrence under conditions of sulfur starvation-induced stress was not considered (47). The current work reveals that the inactivation of *cysR* (encoded by the gene *Synpcc7942_1684*) significantly attenuates the transcription of *nblA* under conditions of 1% CO_2_ and sulfate limitation, suggesting a similar role for CysR (encoded by the gene *Synpcc7942_1684*) in conjunction with NtcA (encoded by the gene *Synpcc7942_0127*) in activating the transcription of *nblA* (encoded by the gene *Synpcc7942_2127*) under sulfate-limiting conditions. Nevertheless, the mechanism underlying the regulation of phycobilisome degradation by CysR (encoded by the gene *Synpcc7942_1684*) under the conditions of high CO_2_ and sulfate limitation remains to be explored.

## Data Availability

All experimental data supporting the findings of this study are available within the article and its supplemental material. The RNA-seq data generated in this study have been deposited in the NCBI Trace Archive database under accession code PRJNA1178830.

## SUPPLEMENTAL MATERIAL

Supplemental material aem.00115-25-s0001.docx Figures S1 to S7; Tables S1 to S6.

ASM does not own the copyrights to Supplemental Material that may be linked to, or accessed through, an article. The authors have granted ASM a non-exclusive, world-wide license to publish the Supplemental Material files. Please contact the corresponding author directly for reuse.
