# Supplementary material for: Sulfate assimilation regulates antioxidant defense response of the cyanobacterium Synechococcus elongatus PCC 7942 to high concentrations of carbon dioxide
Source: Appl Environ Microbiol. 2025 Mar 6;91(4):e00115-25. doi: 10.1128/aem.00115-25 (PMC12016511; doi:10.1128/aem.00115-25)
Supplement: Supplemental material — Figures S1 to S7; Tables S1 to S6. [file aem.00115-25-s0001.docx]

**Supplementary Material for**

**Sulfate assimilation regulates antioxidant defense response of the cyanobacterium *Synechococcus elongatus* PCC 7942 to high concentrations of carbon dioxide**

Yujie Mu, Huiting Chen, Jianwei Li, Pei Han, and Zhen Yan

This PDF file includes:

Figures S1 to S7

Tables S1 to S6

**
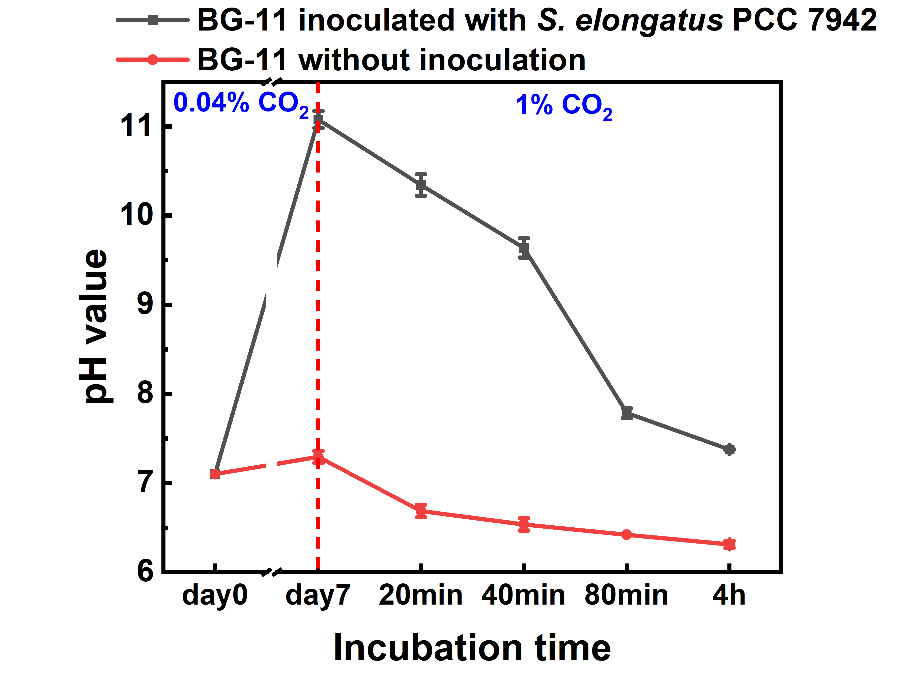
**

**Fig. S1** **The change of pH value of *S. elongatus* PCC 7942 and BG-11 medium upon exposure to 1% CO_2_.**

**
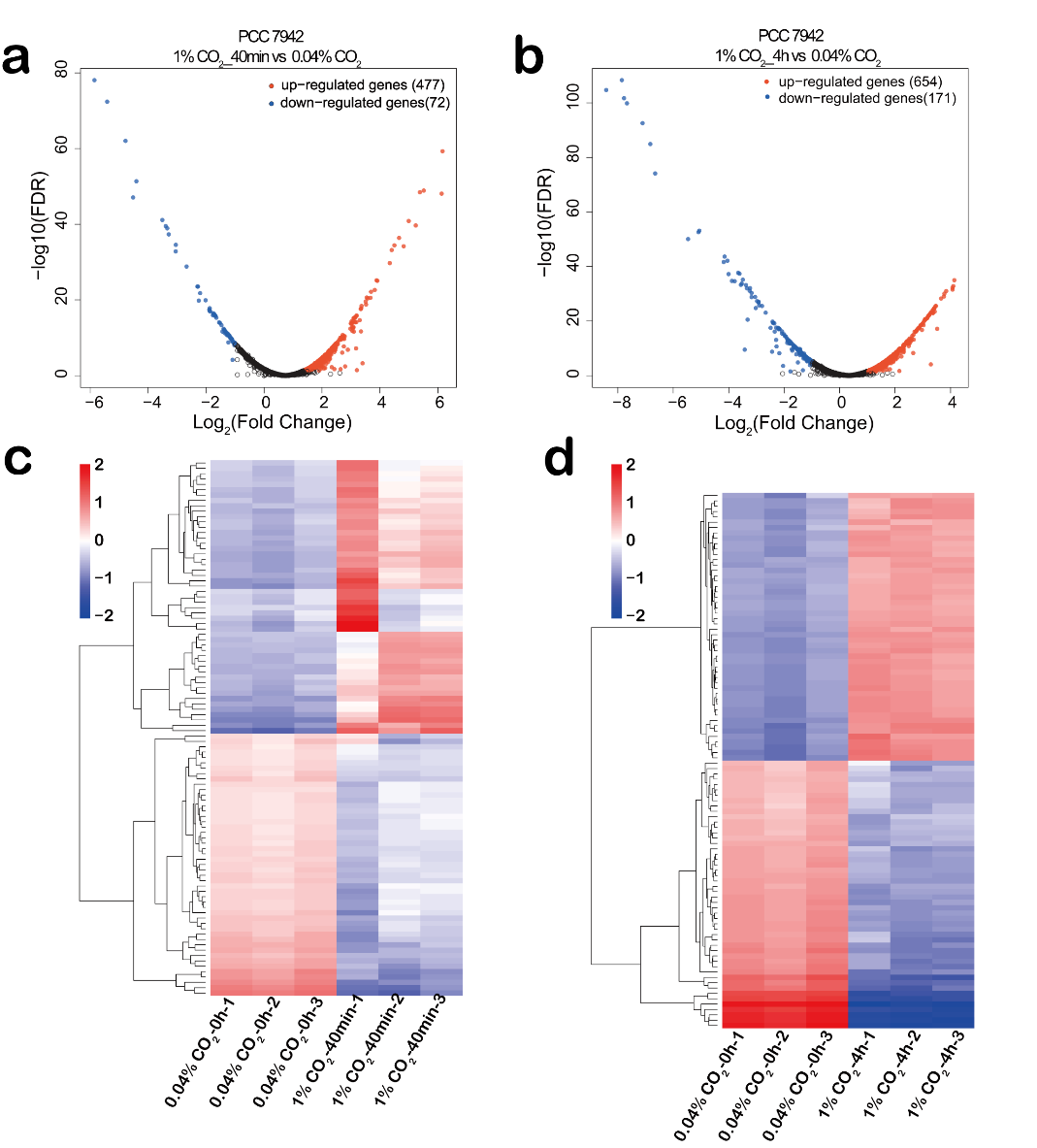
**

**Fig. S2 Transcriptome analysis of *S. elongatus* PCC 7942.** Upregulated and downregulated DEGs of Volcano plot of differentially expressed genes (DEGs) under (a) 1% CO_2_-40min *vs*. 0.04% CO_2_ and (b) 1% CO_2_-4h *vs*. 0.04% CO_2_; Cluster map of DEGs in all groups under (c) 1% CO_2_-40min *vs*. 0.04% CO_2_ and (d) 1% CO_2_-4h *vs*. 0.04% CO_2_.


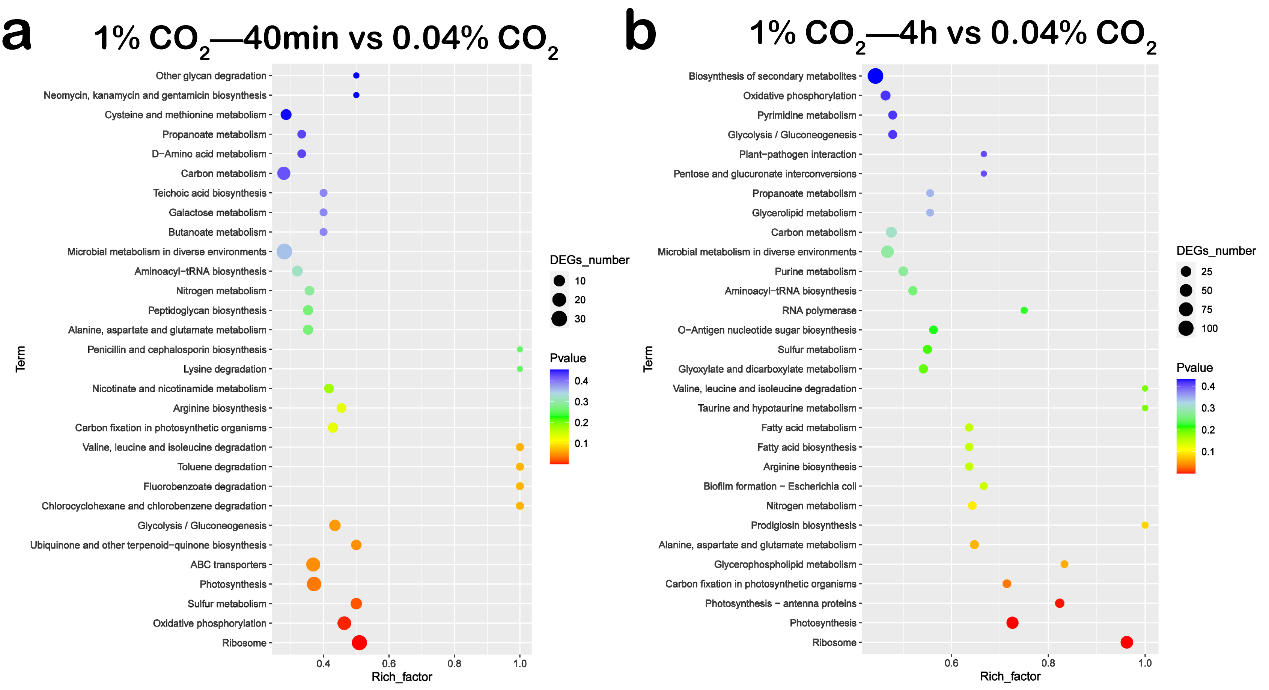


**Fig. S3 KEGG enrichment analysis of differentially expressed genes (DEGs) under (a) 1% CO_2_-40 min vs. 0.04% CO_2_ and (b) 1% CO_2_-4 h vs. 0.04% CO_2_.**


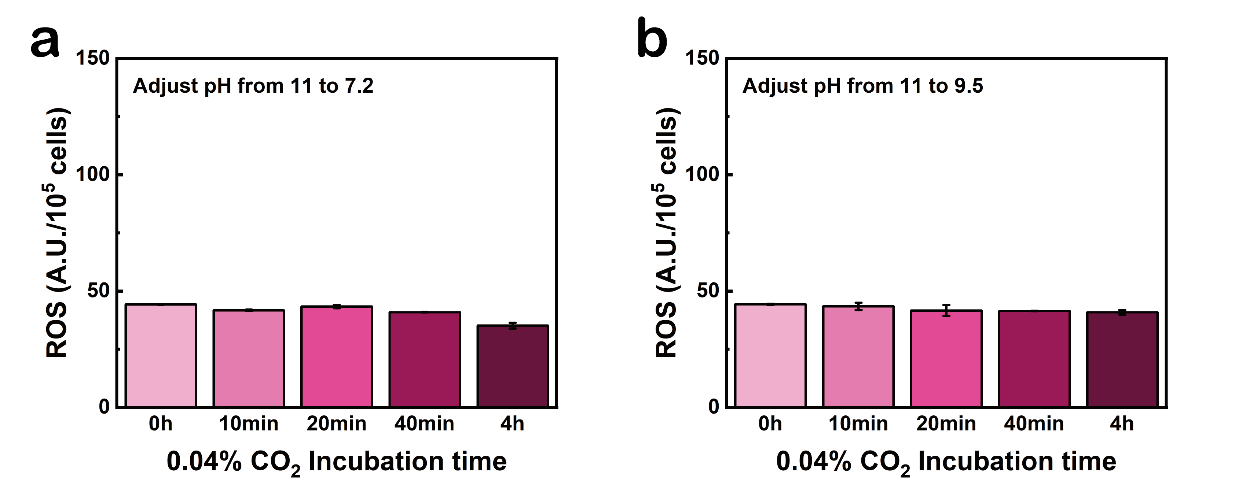


**Fig. S4 Intracellular levels of ROS upon adjusting pH of the culture to 7.2 (a) or 9.5 (b) at day 7.** The pH value of *S. elongatus* PCC 7942 cultures were adjusted by the addition of HCl until an OD_730_ of 0.8 was attained at day 7.


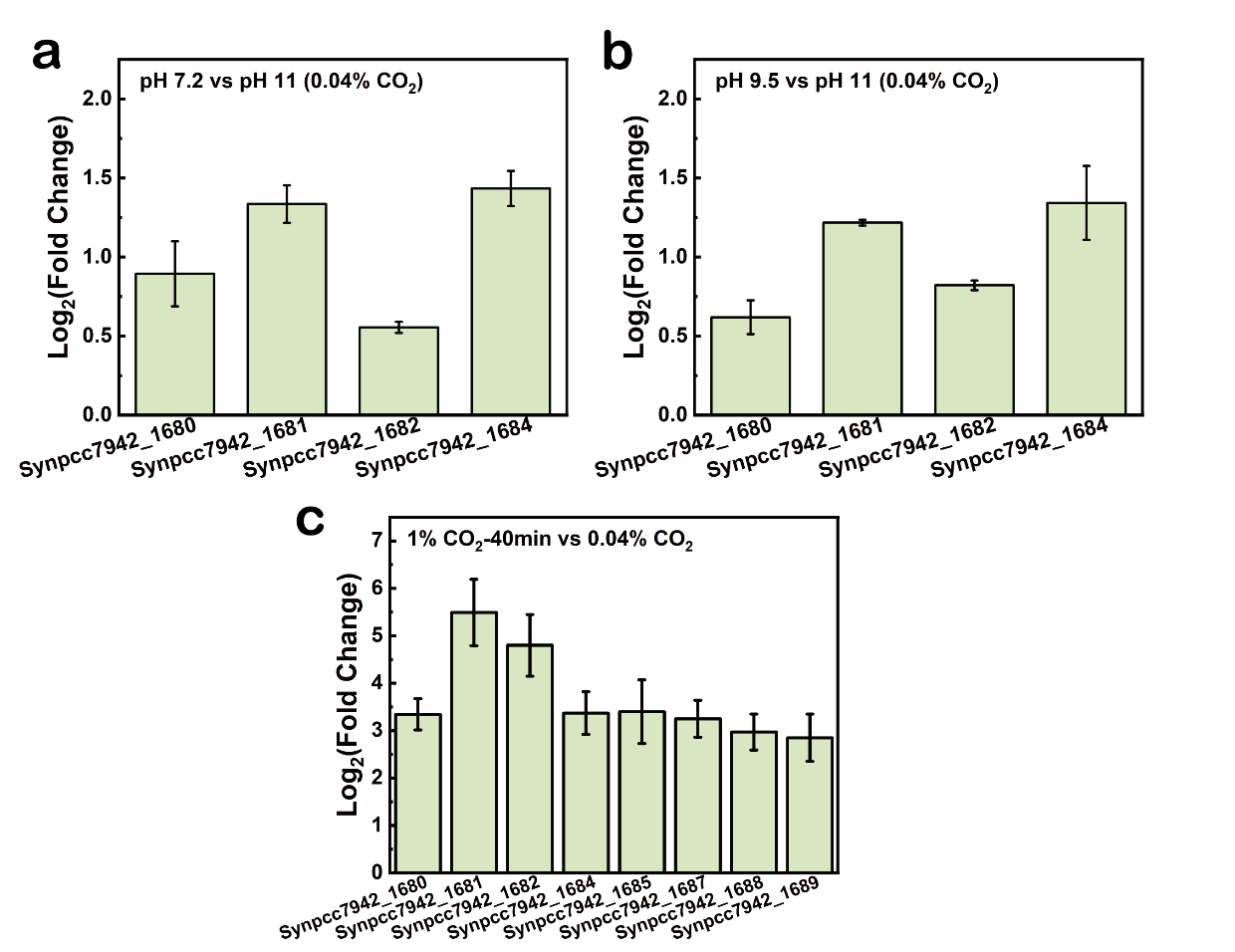


**Fig. S5** **RT-qPCR analysis of *S. elongatus* PCC 7942 upon pH adjustment (a and b) or exposure to 1% CO_2_ (c).** The pH value of *S. elongatus* PCC 7942 cultures were adjusted by the addition of HCl until an OD_730_ of 0.8 was attained at day 7. Abbreviations: *Synpcc7942_1680* encoding **s**ulfate/thiosulfate import ATP-binding protein CysA; *Synpcc7942_1681* encoding sulfate-binding protein SbpA; *Synpcc7942_1682* encoding sulphate transport system permease protein 2; *Synpcc7942_1684* encoding putative transcriptional regulator CysR; *Synpcc7942_1685* encoding sulphate transport system permease protein 2; *Synpcc7942_1687* encoding sulfate ABC transporter, permease protein CysT; *Synpcc7942_1688* encoding sulfate ABC transporter, permease protein CysW.

**
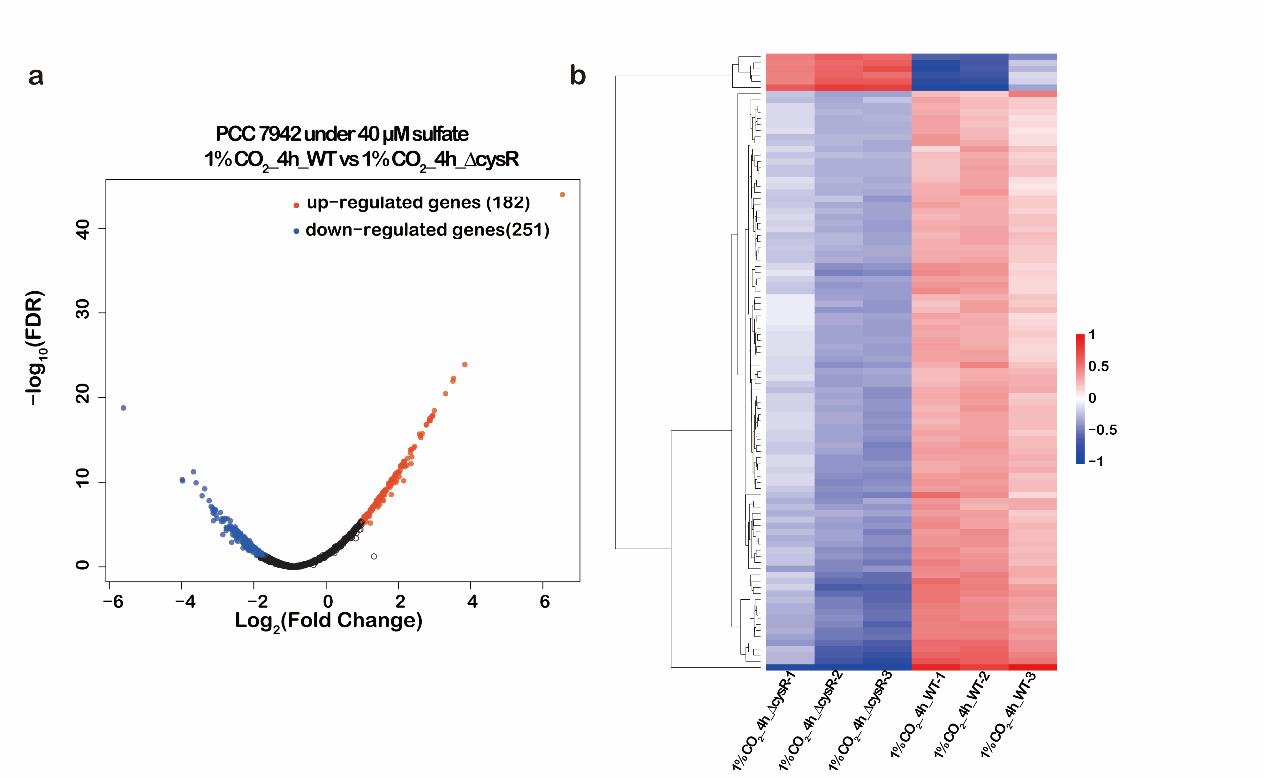
**

**Fig. S6 Transcriptome analysis of WT and Δ*cysR* at the sulfate concentration of 40 μM.** Upregulated and downregulated DEGs of Volcano plot of differentially expressed genes (DEGs) in (a) 1% CO_2_-4 h-WT vs. 1% CO_2_-4 h-Δ*cysR*; Cluster map in all groups under (b) 1% CO_2_-4 h-WT vs. 1% CO_2_-4 h-Δ*cysR*.


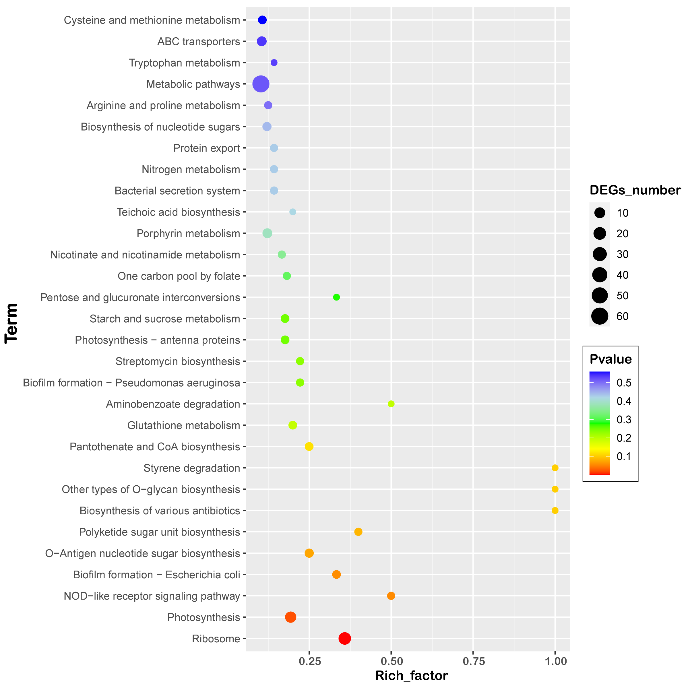


**Fig. S7 KEGG enrichment analysis of differentially expressed genes (DEGs) for 1% CO_2_-4 h-WT vs. 1% CO_2_-4 h-ΔcysR.**

**Table S1.** DEGs in 1% CO_2_-40 min vs. 0.04% CO_2_

| **Functional classification** | **GeneID** | **Function** | **Log_2_FC** | ***p*-value** |
| --- | --- | --- | --- | --- |
| ABC  transporter | Synpcc7942_0351 | ABC-type sugar transport systems  Permease components-like | 1.59057143 | 0.0096 |
|  | Synpcc7942_0526 | ABC-type sugar transport systems  Permease components-like | 1.59057143 | 0.0096 |
|  | Synpcc7942_0815 | ATPase | 2.13240969 | 1.62E-06 |
|  | Synpcc7942_1126 | ABC transporter permease protein | 2.28059873 | 1.98E-08 |
|  | Synpcc7942_1680 | Sulphate transport system  permease protein 1 | 3.88307188 | 6.13E-28 |
|  | Synpcc7942_1682 | Sulphate transport system  permease protein 2 | 4.99236267 | 5.67E-44 |
|  | Synpcc7942_1685 | Sulphate transport system  permease protein 2 | 3.35071748 | 2.65E-20 |
|  | Synpcc7942_1687 | Sulfate ABC transporter,  permease protein CysT | 2.98118982 | 1.11E-14 |
|  | Synpcc7942_1688 | Sulfate ABC transporter,  permease protein CysW | 2.35932443 | 1.68E-08 |
|  | Synpcc7942_1861 | Periplasmic binding protein of ABC  transporter for natural amino acids | 1.59631205 | 0.0042 |
|  | Synpcc7942_1893 | ATPase | 1.87691758 | 5.15E-05 |
|  | Synpcc7942_2106 | Nitrate transport permease | 1.45751769 | 0.0094 |
|  | Synpcc7942_2175 | Transport system substrate-binding  protein | 1.53330354 | 0.0031 |
|  | Synpcc7942_2177 | Integral membrane protein of the  ABC-type Nat permease for  neutral amino acids NatD | 1.56584035 | 0.0041 |
|  | Synpcc7942_2445 | Phosphate binding protein | 1.60095289 | 0.0011 |
|  | Synpcc7942_2573 | Manganese transport system  Membrane protein MntB | 1.67736096 | 0.0009 |
|  | Synpcc7942_0244 | Glycogen/starch/alpha-glucan  phosphorylase | -1.30317605 | 1.45E-13 |
|  | Synpcc7942_0245 | Glyceraldehyde-3-phosphate  dehydrogenase | -1.46971917 | 1.24E-15 |
|  | Synpcc7942_2384 | Pyruvate: ferredoxin  (flavodoxin) oxidoreductase | -1.64666080 | 1.07E-17 |
|  | Synpcc7942_2600 | Protoheme IX  farnesyltransferase | -1.74238244 | 9.83E-19 |
|  | Synpcc7942_2601 | Putative cytochrome  aa3 controlling protein | -1.39619856 | 1.20E-14 |
| Sulfur  metabolism | Synpcc7942_0019 | Sulfite reductase (ferredoxin) | 2.02209112 | 2.35E-06 |
|  | Synpcc7942_0939 | Adenylylsulfate kinase | 2.72907744 | 2.72E-12 |
|  | Synpcc7942_1600 | 5',5'''-P-1, P-4-tetraphosphate  phosphorylase II-like | 2.18388581 | 5.39E-07 |
|  | Synpcc7942_1680 | Sulphate transport system  permease protein 1 | 3.88307188 | 6.13E-28 |
|  | Synpcc7942_1682 | Sulphate transport system  permease protein 2 | 4.99236267 | 5.67E-44 |
|  | Synpcc7942_1685 | Sulphate transport system  permease protein 2 | 3.35071748 | 2.65E-20 |
|  | Synpcc7942_1687 | Sulfate ABC transporter,  permease protein CysT | 2.98118982 | 1.11E-14 |
|  | Synpcc7942_1688 | Sulfate ABC transporter,  permease protein CysW | 2.35932443 | 1.68E-08 |
|  | Synpcc7942_1689 | Rhodanese-like | 3.13913434 | 1.23E-17 |
|  | Synpcc7942_2427 | 3-mercaptopyruvate  sulfurtransferase | 1.85062079 | 4.10E-05 |
| Antioxidative activity | Synpcc7942_0108 | Sulfiredoxin | 3.30178076 | 5.08E-17 |
|  | Synpcc7942_0801 | Superoxide dismutase *sodB* | 1.49316612 | 0.0040 |
|  | Synpcc7942_1214 | Glutathione peroxidase *gpxA* | 3.03647098 | 4.71E-15 |
|  | Synpcc7942_1649 | Rubrerythrin | 5.37922565 | 9.13E-52 |
|  | Synpcc7942_2171 | Starvation induced DNA  binding protein *dps* | 3.02240827 | 9.91E-17 |
|  | Synpcc7942_2309 | Thioredoxin peroxidase | 3.10873056 | 1.27E-17 |
|  | Synpcc7942_2449 | 1-Cys peroxiredoxin | 1.79542860 | 8.54E-05 |

**Table S2.** KEGG enrichment involved in carbon metabolism, photophosphorylation and biosynthetic metabolism responses in 1% CO_2_-40 min vs. 0.04% CO_2_

| **layer1** | **layer2** | **layer3** | **ID** | **Up**  **number** | ***p*-value** | **FDR** |
| --- | --- | --- | --- | --- | --- | --- |
| Metabolism | Energy metabolism | Oxidative phosphorylation | ko00190 | 14 | 0.079454492 | 0.82102975 |
| Metabolism | Energy metabolism | Carbon fixation in photosynthetic organisms | ko00710 | 5 | 0.21543027 | 0.993974091 |
| Metabolism | Biosynthesis of other secondary metabolites | Penicillin and cephalosporin biosynthesis | ko00311 | 1 | 0.235035914 | 0.993974091 |
| Metabolism | Glycan biosynthesis and metabolism | Teichoic acid biosynthesis | ko00552 | 2 | 0.335623039 | 0.993974091 |
| Metabolism | Biosynthesis of other secondary metabolites | Neomycin, kanamycin and gentamicin biosynthesis | ko00524 | 1 | 0.414901721 | 0.993974091 |
| Metabolism | Global and overview maps | Biosynthesis of amino acids | ko01230 | 21 | 0.461969721 | 0.993974091 |
| Metabolism | Lipid metabolism | Fatty acid biosynthesis | ko00061 | 3 | 0.497822918 | 0.993974091 |
| Metabolism | Amino acid metabolism | Lysine biosynthesis | ko00300 | 3 | 0.497822918 | 0.993974091 |
| Metabolism | Glycan biosynthesis and metabolism | O-Antigen nucleotide sugar biosynthesis | ko00541 | 4 | 0.539995811 | 0.993974091 |
| Metabolism | Global and overview maps | Biosynthesis of secondary metabolites | ko01110 | 57 | 0.549219829 | 0.993974091 |
| Metabolism | Biosynthesis of other secondary metabolites | Phenazine biosynthesis | ko00405 | 1 | 0.552530669 | 0.993974091 |
| Metabolism | Biosynthesis of other secondary metabolites | Prodigiosin biosynthesis | ko00333 | 1 | 0.552530669 | 0.993974091 |
| Metabolism | Global and overview maps | Carbon metabolism | ko01200 | 14 | 0.591404279 | 0.993974091 |
| Metabolism | Biosynthesis of other secondary metabolites | Streptomycin biosynthesis | ko00521 | 2 | 0.662730678 | 0.993974091 |
| Metabolism | Metabolism of terpenoids and polyketides | Polyketide sugar unit biosynthesis | ko00523 | 1 | 0.738379348 | 0.993974091 |
| Metabolism | Biosynthesis of other secondary metabolites | Monobactam biosynthesis | ko00261 | 1 | 0.738379348 | 0.993974091 |
| Metabolism | Metabolism of terpenoids and polyketides | Terpenoid backbone biosynthesis | ko00900 | 2 | 0.812488046 | 0.993974091 |
| Metabolism | Metabolism of cofactors and vitamins | Folate biosynthesis | ko00790 | 3 | 0.859554493 | 0.993974091 |
| Metabolism | Amino acid metabolism | Phenylalanine, tyrosine and tryptophan biosynthesis | ko00400 | 3 | 0.859554493 | 0.993974091 |
| Metabolism | Energy metabolism | Photosynthesis - antenna proteins | ko00196 | 2 | 0.935182128 | 0.993974091 |
| Metabolism | Amino acid metabolism | Valine, leucine and isoleucine biosynthesis | ko00290 | 1 | 0.947866913 | 0.993974091 |
| Metabolism | Metabolism of cofactors and vitamins | Pantothenate and CoA biosynthesis | ko00770 | 1 | 0.960174083 | 0.993974091 |
| **SUM** | **143** | **Upregulated DEGs** | **477** | **Ratio** | **143/477=30%** |  |

**Table S3.** KEGG enrichment involved in carbon metabolism, photophosphorylation and biosynthetic metabolism responses in 1% CO_2_-4h vs. 0.04% CO_2_

| **layer1** | **layer2** | **layer3** | **ID** | **Up**  **number** | ***p*-value** | **FDR** |
| --- | --- | --- | --- | --- | --- | --- |
| Metabolism | Energy metabolism | Photosynthesis | ko00195 | 43 | 2.13E-06 | 0.00010641 |
| Metabolism | Energy metabolism | Photosynthesis - antenna proteins | ko00196 | 14 | 0.000433665 | 0.014455507 |
| Metabolism | Biosynthesis of other secondary metabolites | Prodigiosin biosynthesis | ko00333 | 3 | 0.063886361 | 0.999542685 |
| Metabolism | Lipid metabolism | Fatty acid biosynthesis | ko00061 | 7 | 0.098868054 | 0.999542685 |
| Metabolism | Glycan biosynthesis and metabolism | O-Antigen nucleotide sugar biosynthesis | ko00541 | 9 | 0.141522234 | 0.999542685 |
| Genetic Information Processing | Translation | Aminoacyl-tRNA biosynthesis | ko00970 | 13 | 0.152593013 | 0.999542685 |
| Metabolism | Global and overview maps | Biosynthesis of secondary metabolites | ko01110 | 104 | 0.208334702 | 0.999542685 |
| Metabolism | Energy metabolism | Oxidative phosphorylation | ko00190 | 19 | 0.248273021 | 0.999542685 |
| Metabolism | Metabolism of terpenoids and polyketides | Biosynthesis of ansamycins | ko01051 | 1 | 0.4 | 0.999542685 |
| Metabolism | Biosynthesis of other secondary metabolites | Biosynthesis of various antibiotics | ko00998 | 1 | 0.4 | 0.999542685 |
| Metabolism | Glycan biosynthesis and metabolism | Other types of O-glycan biosynthesis | ko00514 | 1 | 0.4 | 0.999542685 |
| Metabolism | Amino acid metabolism | Lysine biosynthesis | ko00300 | 5 | 0.467225733 | 0.999542685 |
| Metabolism | Metabolism of terpenoids and polyketides | Terpenoid backbone biosynthesis | ko00900 | 5 | 0.562046353 | 0.999542685 |
| Metabolism | Metabolism of cofactors and vitamins | Pantothenate and CoA biosynthesis | ko00770 | 5 | 0.562046353 | 0.999542685 |
| Metabolism | Global and overview maps | Carbon metabolism | ko01200 | 24 | 0.590875929 | 0.999542685 |
| Metabolism | Biosynthesis of other secondary metabolites | Novobiocin biosynthesis | ko00401 | 1 | 0.640094712 | 0.999542685 |
| Metabolism | Biosynthesis of other secondary metabolites | Neomycin, kanamycin and gentamicin biosynthesis | ko00524 | 1 | 0.640094712 | 0.999542685 |
| Metabolism | Biosynthesis of other secondary metabolites | Carbapenem biosynthesis | ko00332 | 1 | 0.640094712 | 0.999542685 |
| Metabolism | Global and overview maps | Biosynthesis of amino acids | ko01230 | 33 | 0.662544434 | 0.999542685 |
| Metabolism | Biosynthesis of other secondary metabolites | Monobactam biosynthesis | ko00261 | 2 | 0.66324473 | 0.999542685 |
| Metabolism | Metabolism of terpenoids and polyketides | Polyketide sugar unit biosynthesis | ko00523 | 2 | 0.66324473 | 0.999542685 |
| Metabolism | Metabolism of cofactors and vitamins | Ubiquinone and other terpenoid-quinone biosynthesis | ko00130 | 5 | 0.721356265 | 0.999542685 |
| Metabolism | Global and overview maps | Biosynthesis of nucleotide sugars | ko01250 | 12 | 0.725583569 | 0.999542685 |
| Metabolism | Glycan biosynthesis and metabolism | Peptidoglycan biosynthesis | ko00550 | 6 | 0.736833575 | 0.999542685 |
| Metabolism | Biosynthesis of other secondary metabolites | Streptomycin biosynthesis | ko00521 | 3 | 0.768659071 | 0.999542685 |
| Metabolism | Biosynthesis of other secondary metabolites | Phenazine biosynthesis | ko00405 | 1 | 0.784170496 | 0.999542685 |
| Metabolism | Glycan biosynthesis and metabolism | Teichoic acid biosynthesis | ko00552 | 1 | 0.922444551 | 0.999542685 |
| Metabolism | Global and overview maps | Biosynthesis of cofactors | ko01240 | 41 | 0.957635579 | 0.999542685 |
| Metabolism | Amino acid metabolism | Valine, leucine and isoleucine biosynthesis | ko00290 | 2 | 0.970029116 | 0.999542685 |
| Metabolism | Metabolism of cofactors and vitamins | Folate biosynthesis | ko00790 | 4 | 0.97742643 | 0.999542685 |
| Metabolism | Amino acid metabolism | Phenylalanine, tyrosine and tryptophan biosynthesis | ko00400 | 4 | 0.97742643 | 0.999542685 |
| **SUM** | **373** | **Upregulated DEGs** | **654** | **Ratio** | **373/654=**  **57%** |  |

**Table S4.** DEGs in 1% CO_2_-4 h-WT vs. 1% CO_2_-4 h-Δ*cysR* at the sulfate concentration of 40 μM

| **Functional classification** | **GeneID** | **Function** | **Log_2_FC** | ***p*-value** |
| --- | --- | --- | --- | --- |
| Photo-  synthesis | Synpcc7942_0407 | photosystem I reaction center subunit X | 1.761928899 | 3.47074E-12 |
|  | Synpcc7942_0920 | photosystem I reaction center | 1.968929389 | 1.27459E-13 |
|  | Synpcc7942_1002 | photosystem I reaction center subunit II | 2.953514176 | 3.65977E-21 |
|  | Synpcc7942_1088 | plastocyanin | 1.810820804 | 1.53412E-12 |
|  | Synpcc7942_1322 | photosystem I reaction center subunit IV | 2.097334732 | 1.15306E-14 |
|  | Synpcc7942_1499 | ferredoxin (2Fe-2S) | 1.762453413 | 6.70597E-12 |
|  | Synpcc7942_1679 | photosystem II reaction center protein Psb28 | 1.728063511 | 3.88111E-12 |
|  | Synpcc7942_1882 | psbU; photosystem II complex extrinsic protein U | 1.363698414 | 2.37735E-09 |
|  | Synpcc7942_2245 | psbZ; photosystem II reaction center protein Z | 1.0675538 | 1.06249E-07 |
| Metabolic pathways | Synpcc7942_0056 | perosamine synthetase | 1.110216624 | 6.95042E-08 |
|  | Synpcc7942_0062 | glucose-1-phosphate cytidylyltransferase | 1.829901414 | 1.27357E-12 |
|  | Synpcc7942_0240 | hypothetical protein | 1.564148961 | 1.09911E-10 |
|  | Synpcc7942_0325 | Lc 7.8 apoprotein (core components of the phycobilisomes) | 1.25694844 | 1.49318E-08 |
|  | Synpcc7942_0536 | acyl carrier protein | 1.076061907 | 1.1137E-07 |
|  | Synpcc7942_1088 | plastocyanin | 1.810820804 | 1.53412E-12 |
|  | Synpcc7942_1398 | Cellulose synthase (UDP-forming) | 1.388924508 | 1.08212E-09 |
|  | Synpcc7942_1499 | Ferredoxin (2Fe-2S) | 1.762453413 | 6.70597E-12 |
|  | Synpcc7942_1858 | Heme oxygenase (decyclizing) | 1.645426108 | 4.20819E-11 |
|  | Synpcc7942_1882 | probable photosystem II 12 kD extrinsic protein | 1.363698414 | 2.37735E-09 |
|  | Synpcc7942_2030 | phycobilisome rod-core linker polypeptide | 1.166875207 | 3.48078E-08 |
|  | Synpcc7942_2104 | cyanate lyase | 1.426224307 | 1.66441E-09 |
|  | Synpcc7942_2139 | probable glutathione S-transferase | 1.192436872 | 7.48544E-08 |
|  | Synpcc7942_2151 | Cellulose synthase (UDP-forming) | 1.43082289 | 8.76799E-10 |
|  | Synpcc7942_2245 | putative photosystem II PsbZ protein | 1.0675538 | 1.06249E-07 |
|  | Synpcc7942_2322 | aspartyl/glutamyl-tRNA (Asn/Gln) amidotransferase subunit C | 1.418232529 | 9.82109E-10 |
|  | Synpcc7942_2460 | DNA-cytosine methyltransferase | 1.800228135 | 4.08784E-12 |
|  | Synpcc7942_2604 | cytochrome c oxidase subunit III | 1.458785369 | 6.81547E-10 |
| Antioxidative activity | Synpcc7942_2139 | glutathione S-transferase | 1.192436872 | 7.48544E-08 |
|  | Synpcc7942_0801 | Superoxide dismutase *sodB* | 0.453249151 | 8.35E-04 |
|  | Synpcc7942_1214 | Glutathione peroxidase *gp* | 1.487265164 | 2.10E-09 |
|  | Synpcc7942_2171 | Starvation induced DNA  binding protein *dps* | 0.990453804 | 3.56E-07 |
|  | Synpcc7942_2309 | Thioredoxin peroxidase | 0.307770608 | 0.000930368 |
|  | Synpcc7942_2449 | 1-Cys peroxiredoxin *prx* | 1.278618304 | 1.16786E-07 |

**Table S5.** Primers used for PCR

| **Name** | **Forward sequence（5′→3′）** | **Reverse sequence（5′→3′）** |
| --- | --- | --- |
| *cysR-*up | gattacgccaagcttgcatgatgtctctgcgcctcccttc | cgaatctctcctgcaaaagg |
| *cysR*-down | caccaaggtagtcggcaaataacgagcggccatcctgtcctctg | cagtgaattcgagctcggtaaacatcagatttggaaaagc |
| *SmR*-1 | ccttttgcaggagagattcgatgagggaagcggtgatcg | ttatttgccgactaccttggtg |
| pUC19 | taccgagctcgaattcactg | catgcaagcttggcgtaatc |
| *gp-*up | ccaacctcgcaaaaaaagaacagttggcactacaagctc | tgtctattgccagtccagg |
| *gp-*down | ccttggccttaattggcatc | acgcccattaacagagtgag |
| *SmR*-2 | caccaaggtagtcggcaaataaaatcccaaagcatcacgggac | ttatttgccgactaccttggtg |

**Table S6.** Primers used for RT-qPCR

| **Gene** | **Forward sequence（5′→3′）** | **Reverse sequence（5′→3′）** |
| --- | --- | --- |
| 16s | gcaagcctgacggagcaac | cggacgctttacgcccaaat |
| *cysA* | tcctttctccaatctgctgc | tcctttctccaatctgctgc |
| *cysT* | tgagttggtcttggcgattc | agaccaaaggtcacctcgtag |
| *cysW* | agccgttgatgggagatgtg | agagccgcagtaatgtcttc |
| *cysR* | tttggtgcgctcctctacttg | tttggtgcgctcctctacttg |
| *orf81* | ccttggccttaattggcatc | acgcccattaacagagtgag |
| *sbpA* | aagttaatcgccgtgcagtg | aacggcatagctcactaacg |
| *rhdA* | ttaagccgttgggaaacctg | tgggtgagtaaattgcggag |
| *nblA* | ttccctcagtgttgaacaga | cttggcgaatcatgcctttg |
| *nblR* | ttggctcaacaaatggcagg | ggaatggttaagccgcgatc |
